# Artificial Roughness Encoding with a Bio-inspired MEMS- based Tactile Sensor Array

**DOI:** 10.3390/s90503161

**Published:** 2009-04-27

**Authors:** Calogero Maria Oddo, Lucia Beccai, Martin Felder, Francesco Giovacchini, Maria Chiara Carrozza

**Affiliations:** 1 ARTS Lab - Advanced Robotics Technology and Systems Laboratory, Scuola Superiore Sant'Anna, Polo Sant'Anna Valdera / Viale Rinaldo Piaggio 34, 56025 Pontedera, PI, Italy; E-Mails: oddoc@sssup.it (C.M. O.); f.giovacchini@arts.sssup.it (F. G.); carrozza@sssup.it (M.C. C.); 2 Informatics - Robotics & Embedded Systems, Technical University of Munich / 85748 Garching b. Muenchen, Germany; E-mail: felder@in.tum.de (M. F.)

**Keywords:** MEMS tactile sensor array, bio-inspired sensor, roughness encoding, dynamic touch, static contact imaging

## Abstract

A compliant 2×2 tactile sensor array was developed and investigated for roughness encoding. State of the art cross shape 3D MEMS sensors were integrated with polymeric packaging providing in total 16 sensitive elements to external mechanical stimuli in an area of about 20 mm^2^, similarly to the SA1 innervation density in humans. Experimental analysis of the bio-inspired tactile sensor array was performed by using ridged surfaces, with spatial periods from 2.6 mm to 4.1 mm, which were indented with regulated 1N normal force and stroked at constant sliding velocity from 15 mm/s to 48 mm/s. A repeatable and expected frequency shift of the sensor outputs depending on the applied stimulus and on its scanning velocity was observed between 3.66 Hz and 18.46 Hz with an overall maximum error of 1.7%. The tactile sensor could also perform contact imaging during static stimulus indentation. The experiments demonstrated the suitability of this approach for the design of a roughness encoding tactile sensor for an artificial fingerpad.

## 1. Introduction

Artificial tactile sensors which aim to mimic human discrimination capabilities should encode information correlated with the stimulus spatial features, with its motion dynamics as well as with contact mechanics. Roughness is a fundamental feature for texture perception [1,2,3], which has been associated with the spatial modulation of the used stimuli (i.e. “surface coarseness”) [4]. In experiments on human perception of tactile roughness the type of used surfaces often have patterns (gratings or rising dots) with features that can be independently varied in size and spacing [5,6]. This way, unlike for natural surfaces in which the spatial pattern features vary randomly, the physical characteristics of the explored surface, on which roughness perception is based, can be studied and identified.

The physical determinant of perceived roughness is not yet fully understood [2,4] and there is a varied set of spatial features that should be taken into account for studies on roughness perception (e.g., using ridged stimuli: groove width, ridge width, ridge orientation, ridge height, material compliance, surface lubrication and fine finishing, etc.). In human psychophysical experiments, for example, some groups highlighted the presence of a relatively narrow region where the sense of roughness increases together with the groove width of ridged stimuli, followed by a flattened perception in case of very coarse gratings (up to 8.5 mm of groove width) [7]. In parallel to this, using embossed dots, some researchers presented monotonic functions of roughness and dots spacing [5], while an inverse “U” shape was shown in [8].

Considering dynamic exploration of extremely fine textures, various researchers showed that humans can detect even up to microtextures [9], highlighting the role of fingerprint ridges as vibration promoters [10] and considering the Pacinian Corpuscles as vibration detectors [11]. Some groups joined the Katz's *duplex theory* considering vibrations useful for revealing fine forms, and a spatial mechanism (i.e. the static image of the contact between the texture and the finger) as the basis for coarse surfaces roughness perception [12]. Importantly, in the last decade, other groups proposed and gave evidence to a unified peripheral neural mechanism highlighting the role of SA1 afferents with respect to the other mechanoreceptors [13,1].

The understanding of the neural mechanisms underlying roughness encoding is in progress, however evidence was given to the fact that temporal frequency changes of tactile information play a major role in roughness perception in humans [5]. Finite element analyses using human finger model during dynamic touch showed that spatial information of the textured surface are related to temporal frequency changes at the position of tactile receptors [14]. In touch activities, if humans have the ability to estimate somehow the relative hand velocity *v* between the textured surface and the exploring finger, the spatial period Δ*p* of the surface can be perceived by detecting the temporal frequency of the vibration [15], such that:

(1) $$f=\frac{v}{\Delta p}$$

The findings and debate of researchers on human touch are directly linked with the development of artificial tactile sensors, which is one of the chief challenges in robotics. Many technologies have been investigated and can be analysed in comprehensive reviews on the topic [16,17]. For the above reasons, with regards to the many reported efforts to reproduce human capability to detect texture, the developed sensors were mainly based on the analysis of the vibration gathered during dynamic exploration or on the contact imaging [18] by means of static indentation. An approach was to develop a finger-like multilayered texture sensor integrating five strain gauges for identifying the difference in roughness, softness and frictional properties of various materials [19]. Employing such device, the texture information of a surface was quantitatively detected by estimating the vibrational frequency excited by indenting and sliding a periodic stimulus with spatial wavelength in the millimiters range [20]. A similar method was previously shown in [21] for finer surfaces. Another noticeable solution was presented in [22], where a spatial filter function was used adaptively depending on sensor-stimulus relative motion parameters, thus pointing out the centrality of a spatio-temporal approach in tactile sensing. Hosoda and colleagues developed a soft fingertip with randomly distributed strain gauges and PVDF films at different depths [23], allowing for discrimination of five different types of materials. Other recent biomimetic fingertips focused on the transduction properties, which could be either acoustic [24] or electrical [25], of the packaging materials for converting the surface features of the explored textures into recorded vibrations. Finally, one of the most recent developments is represented by a high-resolution thin film sensor built by Maheshwari and Saraf [26] by means of a layer-by-layer self-assembly technique, that responds to an applied force either with electroluminescent emissions or with a change in current density. A charge-coupled device (CCD) camera was used to capture the electroluminescent emissions from the sensor providing imaging stress distribution with spatial resolution of about 40 μm.

In this work, the investigated artificial tactile sensor integrates a MEMS array having a number of sensing elements (16 channels in about 20 mm^2^, i.e. 0.8 channels/mm^2^) similar to the innervation density of *Slowly Adapting type 1* (SA1) mechanoreceptors in the hand (about 1 unit/mm^2^) [27]. The technological approach is based on a 3D MEMS core unit [28] with a soft and compliant packaging. As previously demonstrated, the microsensor can be integrated with a packaging architecture resulting in a robust and compliant tactile sensor for application in artificial hands, while sensitive enough to detect slip events, showing that silicon based tactile sensors can go beyond laboratory practice [29].

In the long term, the presented artificial approach aims, on one side, at developing a device capable of mimicking the texture discrimination properties of the human hand and which can be integrated in an anthropomorphic artificial hand, while on the other it is intended as an artificial model to be used as a test bench for neuroscientific hypotheses describing the mechanisms of roughness perception. This long term objective gets inspiration from the above mentioned work of Yoshioka and colleagues [13], in which it was shown that spatial variation in the firing rates of SA1 units-only can account for roughness perception even when the explored texture is finer than the SA1 innervation density.

In order to go in such direction, the specific objective of the current work was to gather the vibrations which are supposed to be the basis for the encoding of roughness in dynamic touch, as well as to perform static imaging of the contact with the same array of tactile sensors. The present experimental analysis evaluates whether there is a substantial processing advantage in using more than one output of the array for finding out the common principal frequency produced during dynamic stimulus presentation. This way, by merging the estimation of the common frequency detected by more than one sensor unit together with the knowledge of the sliding velocity of the applied stimulus, texture related features could be extracted. The suitability of the sensor for both static contact imaging and vibration detection was evaluated by means of an experimental protocol containing both motionless and dynamic contact phases involving forces and velocities in the range of those used by humans in discriminative touch.

The paper is organized as follows. In Section 2 the design of the sensor array is shown, describing the elementary MEMS unit, the packaging and the readout electronics. In Section 3 the experimental protocol and the used data analysis methods are presented. Section 4 shows the experimentation with the array prototype, which has been carried out with ridged stimuli sliding at constant velocity and regulated normal force after and before a static indentation phase. Finally, results are discussed in Section 5 and future work insights are given in the Conclusions.

## 2. Materials

### 2.1. MEMS Sensor Array

**Figure 1.**
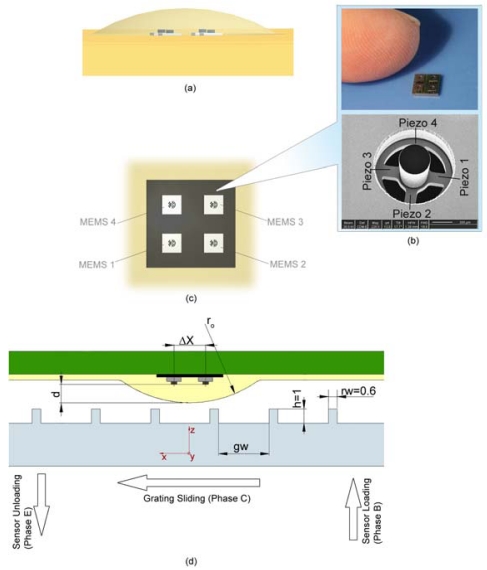
(a) 3D design of the tactile sensor array. (b) Top: The 2×2 MEMS array compared with human finger; bottom: a FIB image of the MEMS sensor. (c) Top view of the sensor array. (d) Schematic showing a cross section of packaging design and grating dimensions. Groove width *gw* ranged from 2.0 mm to 3.5 mm (see Table1), while ridge height *h* and ridge width *rw* had fixed values indicated (in mm) in figure. The phases of the experimental protocol are also indicated.

The elementary cell of the array was the 3D MEMS sensor described in [28], shown in Figure 1, which has a high aspect ratio 3D structure (1.5 mm×1.5 mm×625 μm). In the bare configuration of the sensor, the cylindrical mesa, located at the center of the cross-shape tethers, transmits an externally applied force to the sensor inducing stresses in the four tethers where four p-type piezoresistors are implanted. The fractional change in resistance ΔR/R of each piezoresistor of the microsensor is proportional to the longitudinal and the transversal stress components, while the design of the sensor is such that the transversal stress component in the implanted piezoresistors is neglectable with respect to the longitudinal one. In the current experimentation four microsensors were bonded on a silicon carrier chip connecting the 9 NiAu pads of each microsensor by means of a micro-soldering paste by using flip-chip bonding method. As stated in the Introduction in this study attention was paid in developing an array with a density of sensing elements that could be compared to the innervation density of Slowly Adapting type 1 (SA1) mechanoreceptors in the hand (≈ 1 unit/mm^2^) [27]. The tactile sensor array, depicted in Figure 1, had 16 channels as total tactile sensor outputs. It had a pitch of 2.3 mm (indicated by ΔX in Figure 1) for technological reasons, i.e. mainly because of the operation room needed for the flip-chip bonding method and layout of the carrier chip. The resulting area of the sensing array was of 21.16 mm^2^ inscribing each MEMS unit inside a square of area 5.29 mm^2^. The silicon carrier chip was wire bonded by means of 25 μm Al wires to a Printed Circuit Board (PCB) in order to connect the array to the external instrumentation. The perimeter of the array was secured with a two component epoxy glue in order to protect the wire bonding and to improve the stability of the silicon carrier chip.

The MEMS tactile array was packaged with a synthetic material (as explained in the next section) that mechanically filters the external applied load and creates a distribution of stresses in the new configuration of microsensor and packaging, with respect to the externally applied stimulus. For the investigation reported in the present work, the outputs of the piezoresistors (i.e. the sensing elements) have been analyzed directly for their dynamic behaviour, whilst they have not been used to extract the three components of an applied force. This avoided to address the calibration of each MEMS before packaging, as done with this device in [30], or after packaging as for example performed in [31] with a different sensor together with the introduction of an analytical model for point contact loads.

### 2.2. Packaging

The packaging of the bare silicon sensors array was developed so that the resulting tactile sensor could have compliance and softness characteristics inspired to those of the human fingerpad. Previous investigations for the application in an anthropomorphic artificial hand were considered, in which it was demonstrated that it is possible to integrate the silicon microsensor in a soft and compliant, but robust packaging [29]. In particular, the round shape of the packaging of the array was chosen based on the anthropomorphic features of the distal phalanx of the cybernetic hand CyberHand [32,33]. In parallel, a suitable curved geometry was identified in order to increase the portion of load gathered by the sensors in case of contact with a planar textured surface, as pointed out in [34]. As shown in Figure 1, the dimensioning parameters for the packaging where *r_0_* and *d*, which were set to 8 mm and 1.3 mm, respectively, for obtaining adequate sensitivity as well as partially overlapping sensing ranges between *nearest-neighbour* MEMS units and acceptable low-pass spatial filtering effect [35] with respect to the used stimuli.

The 2×2 array was packaged with polyurethane (Poly 74-40, PolyTek, USA) and an outer thin protective layer of polyimide having thickness of 0.05 mm and shore A 82 hardness (ST1882, Stevens Urethanes, USA) in order to prevent the inner packaging from wearing. In fact, preliminary trials demonstrated that the ridged stimuli can damage the packaging of the array. In the present study, a type of polyurethane with shore A 40 hardness (instead of the previous shore A 45 [29]) was used attempting a step forward in human finger mimicry. Moulds hosting the array were built with rapid prototyping resin using a 3D printer, and the liquid part A and part B were poured immediately after being mixed and degassed. The polyimide layer was applied after polyurethane curing and showed excellent adhesion provided that the air between the cover and the thin sheet was removed. Moreover, the protective layer was secured by means of a frame also built in rapid prototyping resin.

### 2.3. Readout Electronics

**Figure 2.**
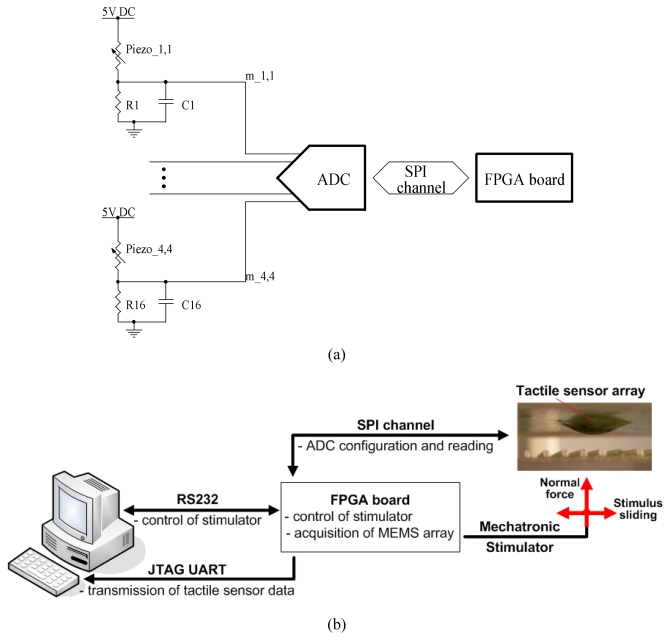
Schematic view of the readout electronics (a). Block diagram of the overall experimental setup (b).

Each piezoresistor was connected in series to a surface mount resistor (R1 … R16) located on the designed PCB, as shown in Figure 2(a). The values of R1 … R16 were all set to 820 Ω, which is close to the mean resistance of the piezoresistors of the 2×2 array, thus almost achieving sensitivity maximization from the quarter bridge voltage divider. The used quarter bridge topology produces a variation of the acquired voltage proportional to the fractional change in resistance of each piezoresistor. Capacitors (C1 … C16, all having capacitance of 1 μF) were placed in parallel to each completing resistor, resulting in a low-pass single pole filter at about 390 Hz (i.e. $\frac{1}{2\pi (R_{\text{piezo}}//R)C}\approx \frac{1}{\pi R_{\text{piezo}}C}$) for reducing the noise level at frequencies outside the band of interest. The piezoresistor-resistor arms were supplied by means of a 5V DC regulated voltage, and the node between each piezoresistor and the completing resistor was directly acquired without pre-amplification by means of a 16-channel 24-bit Analog to Digital Converter (ADS1258, Texas Instruments). Each channel was sampled at a frequency of 241 Hz, which could be varied via software up to 24.7 kHz selecting a subset of channels and changing the conversion options of the used ADC. Considering the chosen sampling frequency and the cut-off of the RC low-pass filter, the fulfillment of the Nyquist theorem for aliasing avoidance mainly relied on the expected baseband properties of the gathered signals (refer to Figures 4 and 5 in the following for a qualitative validation of such assumption). Digital data transfer between the ADC and the acquisition system was performed by means of SPI protocol. The data acquisition system was based on Field Programmable Gate Array (FPGA) technology (CycloneII, Altera) and had a 64 bit hardware timer running at 50 MHz, so that the acquisition of each channel had a time reference with resolution of 20 ns and practically unlimited length. Acquired data was buffered by a soft-core processor (NiosII, Altera) instantiated onboard the FPGA and transmitted at the end of each session to a Personal Computer with JTAG UART protocol, as shown in Figure 2(b). The storage of data was allowed within the Nios II Integrated Development Environment by enabling the option “Filing System to open files on the PC” in the Altera Host Based File System.

## 3. Methods

### 3.1. Experimental Protocol

The packaged array was mounted on a mechatronic tactile stimulator capable of indenting the sensor with force feedback control and stroking a stimulus over it with precise position control. The configuration of the array/stimulus interface and the experimental protocol are reported in Figure 1(d) while Figure 2(b) shows a diagram of the overall experimental set-up.

Four types of stimuli were built with rapid prototyping resin material, with spatial periods Δ*p* varying from a minimum of 2.6 mm to a maximum of 4.1 mm, as detailed in Table 1.

In order to evaluate whether the sensor outputs could be processed for automatically recognizing the instant of contact, data acquisition started prior to the phase during which the stimulus contacted the sensor array (phase A). In a second phase of the experiment, the stimulator was commanded to contact the tactile sensor (phase B). The sensor array was loaded setting at 1 N the reference of the normal force feedback controller given that such value is in the middle of the force range used by humans in fine forms discrimination during active dynamic touch experience [36].

The loading resulted in a contact spike in the signals gathered from the MEMS array. The target force level was held for 1 s. After that, the sliding of stimulus started (phase C) along the *x*-axis [piezo1-piezo3 direction of Figure 1(b)] while maintaining enabled the force feedback controller, thus obtaining a stimulation with normal force held at 1 N and tangential force depending on the contact mechanics and on the motion dynamics.

Three different translational velocities (15 mm/s, 30 mm/s and 48 mm/s) of the stimulus were chosen for overlapping with the range commonly used in related neurophysiologic studies [37]. The direction of motion (along the *x*-axis as shown in Figure 1) was always the same, as well as the sign of velocity and the starting absolute position. The sliding was applied for 60 mm, providing dynamic stimulations of 4 s, 2 s or 1.25 s depending on the applied velocity. At the end of the sliding motion there was a steady state of 1 s at 1 N (phase D) and, finally, the tactile sensor array was unloaded (phase E). The initial and final static phases of the protocol were performed with repeatable conditions in order to enable analyses on static imaging capabilities of the sensor in addition to the dynamic behaviour investigation.

### 3.2. Common Frequency Detection

#### Preprocessing

**Figure 3.**
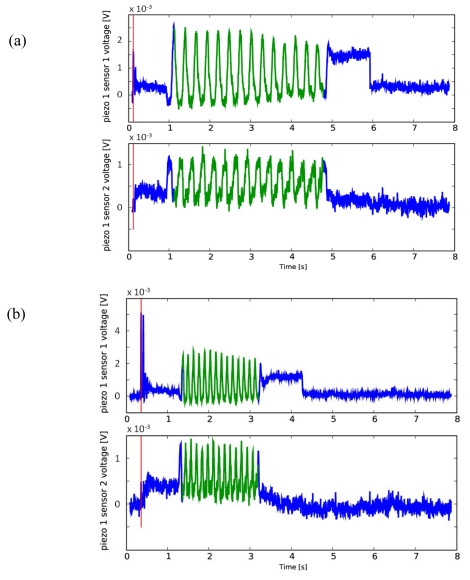
Automatic contact detection applying Equations (2) and (3) and selection of the stimulus sliding phase (phase C, indicated in green in the plots) by means of Equations (4) and (5) using piezoresistor 1 of MEMS sensors 1 and 2 of the array. The plots refer to data series 12 (a) and 16 (b), where a grating of 4.1 mm spatial periodicity was applied with translational speeds of 15 mm/s and 30 mm/s, respectively, according to Tables 2 and 3. The red line marks the detected spike, blue data are cropped for common frequency analysis.

During the sliding of the periodic ridged stimulus over the packaged sensor array (phase C; see Figure 1), the output signal *m_i,j_* from the *i-th* piezoresistor of the *j-th* MEMS unit of the 2×2 array clearly showed a principal frequency component *f*, while the contact and the unloading operations could be revealed by the first spike and the last step in the outputs, as shown in Figure 3.

Defining as *v* the sliding velocity of the grating, the relationship reported in Equation (1) is expected for *f*.

Referring to Figure 1 (d) and Table 1, the spatial period Δ*p_k_* of the of the *k-th* grating is given by the sum of the groove width and of the ridge width, thus: Δ*p_k_ = gw_k_ + rw_k_*.

In order to be able to detect the common frequency between all the fitting curves of the output signals, attention was paid in respecting the Nyquist condition for the sampling frequency $\left(f<\frac{f_{C}}{2}\right)$ with a safety factor, such that at least 13 samples per period were guaranteed even in the worst case stimulation conditions (i.e. minimum grating periodicity Δ*p_k_* and maximum speed of the stimulus, as shown in Table 2).

To ensure data quality, a simple procedure was implemented to remove prior to processing data that was not useful for the dynamic analysis of the recorded signals. The redundancy in the system was used by jointly observing the outputs of two piezoresistors from different MEMS sensors. Since the contact spike mentioned in Section 3.1 was less pronounced for some piezoresistors than for others, the best defined spike was extracted from either one of the two time series.

To this end:

(2) $$s=\underset{j}{\arg\max}\left(\max\left(m_{i,j}\left(t\leq t_{\text{init}}\right)\right)-\text{median}\left(m_{i,j}\left(t\leq t_{\text{init}}\right)\right)\right)$$

selects the sensor *s* whose data was used for initial spike detection. Here, *t*_init_ = 0.8s is a time threshold before which the spike is expected to appear. The location of the spike *t*_spike_ is then detected by:

(3) $$t_{\text{spike}}=\underset{t}{\arg\max}\left(m_{i,s}\left(t\right)\right)$$

and used for both time series. According to the measurement protocol, the movement starts at *t* = 1.0s after the spike, and ends after 60mm of stimulus have been traversed. The effective sample length was set to *L*_eff_ = 55mm for pre-processing operations, in order to avoid introducing invalid data in case of inherent timing variations. The start and the end of the valid range thus were:

(4) $$t_{\text{start}}=t_{\text{spike}}+\Delta t$$

(5) $$t_{\text{end}}=t_{\text{start}}+\frac{L_{\text{eff}}}{v}$$

with *ν* denoting the sliding velocity of the grating.

Ten different combinations of sliding velocity and grating periodicity were formed, as detailed in Section 4, Table 3. Four measurement runs of the sensor array were carried out for each combination, yielding a total of 40 runs. In the following, these data series are referred by number, with run 1 to 4 belonging to combination one, run 5 to 8 to combination 2 and so forth. Figure 3 shows typical outputs of the procedure, which performed flawlessly on all 40 data sets.

An optional pre-processing step consisted in chopping the time series into time windows of size *w*. In most practical applications with gradually or abruptly changing surface characteristics, a trade-off will have to be found between the response delay given by the finite window size, and the accuracy of retrieval. Here this is investigated with non-overlapping windows to minimize redundancy, while in practice one could probably use strongly overlapping windows and thus higher update rates, if enough computation power is available.

#### Fast Fourier Transform

The most important prerequisite for advanced use of the developed sensor array was to establish a robust retrieval procedure for the fundamental spatio-temporal frequency of the system. As a first step, the two selected piezoresistors voltage time series underwent a Fast Fourier Transform (FFT) separately, to find a first guess for the fundamental frequency *f*, namely at the maximum of the periodogram. Note that by selecting the maximum peak as the fundamental frequency using naïve Fourier analysis, a discretization error of up to:

(6) $$\frac{1}{2}\Delta f=\frac{1}{2}\frac{f_{C}}{N}=\frac{1}{2w}$$

occurred, where *f*_C_ = 241 Hz was the channel sampling frequency and *N* the number of data points in the time window. The first guess magnitude of the fundamental oscillation was also difficult to read from the spectrum, because it would have to include contributions from the slopes surrounding the central peak. The solutions implemented in the following overcame this inconvenience. While it would certainly improve accuracy to average the contributions surrounding the fundamental frequency peak and/or to take into account overtones that often can be seen in the spectra, this procedure would involve several heuristic decisions about thresholds and boundaries.

#### Least squares fitting

In order to overcome to the discretization problem mentioned above, during dynamic stimulation each sensor output was fitted with a sine wave by using Equation (7):

(7) $$m_{i,j}\approx B_{i,j}+A_{i,j}\cdot \sin\left(2\pi f\left(t-t_{i,j}\right)\right)$$

where:

– *m_i_,_j_* is the signal obtained from the *i-th* piezoresistor of the *j-th* MEMS unit of the 2×2 array;
– *B_i,j_* and *A_i,j_* are the offset and the amplitude of the sine waves used for fitting each *m_i,j_*;
– *t_i,j_* is an offset time which well fits the sine waves with data acquired during the exploration phase;
– *f* is the common principal frequency coming out from the output signals using the analysis described below; observe that *f* is expected to be the same for all the outputs of the sensor array.

Therefore, a simpler second step was chosen, where a function of the form:

(8) $$h(t,f,A_{i,j},B_{i,j},t_{i,j})=B_{i,j}+A_{i,j}\sin\left(2\pi f(t-t_{i,j})\right)$$

was defined to be fitted to each channel's time domain data (see also Equation (7)). This was done by performing a gradient descent on the error function (considering piezoresistor 1 of MEMS sensors 1 and 2) overall the runs of a same combination of grating and velocity:

(9) $$E=\sum_{k}\left({\left(h(t_{k},f,A_{1,1},B_{1,1},t_{1,1})-m_{1,1}(t_{k})\right)}^{2}+{\left(h(t_{k},f,A_{1,2},B_{1,2},t_{1,2})-m_{1,2}(t_{k})\right)}^{2}\right)$$

with *k* running over all data points in the chosen time window, *t_k_* the sampling instants and *m_i,j_*(*t_k_*) the signal obtained from piezoresistor *i* of unit *j* of the array. Thus there were seven fitting parameters: *A*_1,1_, *A*_1,2_, *B*_1,1_, *B*_1,2_, *t*_1,1_, *t*_1,2_, and *f*. The purpose of this procedure was to both remove the discretization errors of the FFT, and introduce a priori information, because all the sensors were dragged over the same physical surface at the same speed, and then the same fundamental frequency was expected. It is possible to extend this method to include all valid piezoresistor readings from all sensors, if additional accuracy is required. To test this retrieval procedure, data from all measurement series was processed, averaging over the four measurements of each configuration of grating width and velocity. The data window width *w* was varied from 0.2 s to 1.0 s. For each *w*, the start of the time window was stepped through from *t_start_* to *t_end_-w* in steps of 50 ms.

### 3.3. Error Parameters and Repeatability

The RMS error between the estimated frequency and the nominal one was used as a quality index for comparing the FFT results with the fitting procedure described above, i.e.:

(10) $${ɛ}_{\text{method}}(C,w)=\sqrt{\frac{1}{n\cdot q}\sum_{n,q}{\left(f_{\text{estimated}}(n,q)-f_{\text{nominal}}(C)\right)}^{2}}$$

where the subscript *method* may be FFT or LSq depending on the usage of Naïve Fourier analysis or time domain least squares fit, respectively, for estimating the principal frequency. Moreover, *n* loops over all time windows in a measurement run, and *q* over all four runs belonging to parameter combination *C* and window size *w*. Also, a relative error parameter was used by dividing the RMS error of Equation (10) by the nominal frequency *f_nominal_*(*C*) and expressing the result as a percentage.

Repeatability was checked by pairwise cross-correlation of the measurement timeseries of piezoresistor *i* of MEMS unit *j* during the stimulus sliding phase (cf. Figure 3) of the four runs sharing one parameter combination *C*, and averaging the results. Hence, with *l* and *p* denoting two of those four runs:

(11) $$r_{i,j}(C)=\frac{1}{12}\sum_{l\neq p}\left(\frac{1}{k-1}\sum_{t=t_{a}}^{t_{a+k}}\left(\frac{m_{i,j,l}(t)-{\bar{m}}_{i,j,l}}{\sigma_{i,j,l}}\frac{m_{i,j,p}(t)-{\bar{m}}_{i,j,p}}{\sigma_{i,j,p}}\right)\right)$$

is the average Pearson cross-correlation coefficient. Measurements *m* had to be shifted by up to half a cycle relative to each other to account for phase differences due to the lack of synchronization between the starting of data saving and the starting of stimulation between different runs. Therefore, the inner sum runs over the remaining overlap region *t_a_* to *t_a+k_*, for which the mean signal *m̄* and the standard deviation *σ* are calculated.

## 4. Experimental Results

This Section reports the experimental results obtained indenting and sliding the used ridged stimuli according to the parameters given in Table 2. The first part reports the preliminary naïve Fourier analysis which was performed in the process for establishing a robust retrieval procedure for the principal frequency induced by the grating spatial periodicity and sliding speed. Those preliminary results, as expected, were affected by considerable and oscillatory discretization errors depending on the chosen observation window. The second part shows the results with the proposed least squares fitting procedure, which guaranteed very high accuracy and quite fast error convergence increasing the observation window. Furthermore, the results with the least squares fitting are compared with the Fast Fourier Transform ones. Qualitative and quantitative evidence of data repeatability is given in the third part. Finally, the last subsection concerns results on the static imaging capabilities as another major feature of the designed sensor.

### Fast Fourier Transform

**Figure 4.**
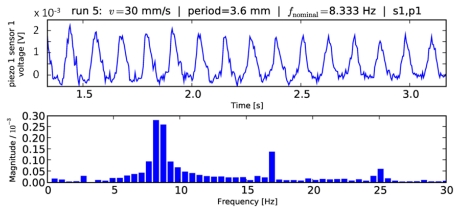
Naïve Fourier analysis (lower plot) over the full length of a typical dataset considering a single channel of a sensor unit of the array (upper plot). The maximum Fourier peak is selected as a frequency estimate, which leads to a discretization error of up to half a bar width (cf. Equation (6)) if the true frequency happens to lie in between two bars.

**Figure 5.**
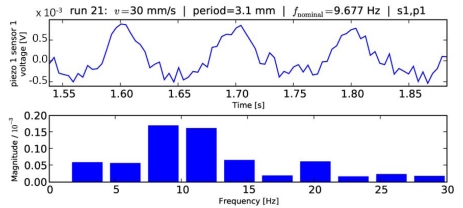
As Figure 4, but for a different data series and a narrower window of 0.35 s, showing higher discretization error with naïve Fourier analysis.

The preliminary naïve Fourier analysis showed a considerable discretization error, according to Equation (6). This error can be quite significant for small windows, since the fundamental frequencies considered lie in the order of 1 to 10 Hz in this experiment. Figure 4 shows a typical Fourier analysis covering the full range of a sliding measurement (phase C, depicted in Figure 1). As a rather extreme example, the results using FFT with a 0.35 s time window analysis can be seen in Figure 5.

### Least Squares Fitting

**Figure 6.**
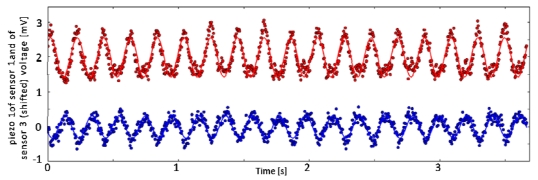
Result of the least squares fitting procedure considering piezoresistor 1 of MEMS sensor 1 (blue line) and piezoresistor 1 of MEMS sensor 2 (red line, shifted for easing the graphical representation) with a maximum width time window. The plot refers to data series 20, where a grating of 3.1 mm spatial periodicity was applied with translational speed of 15 mm/s, according to Tables 2 and 3.

**Figure 7.**
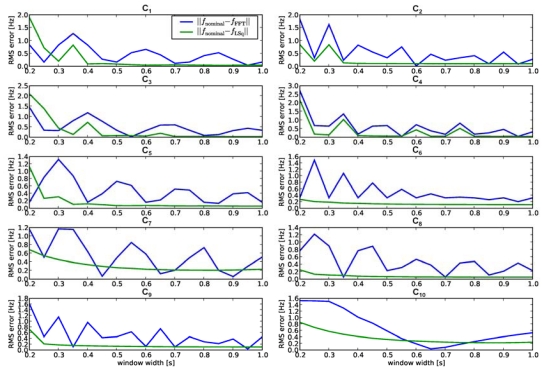
Frequency estimation errors per combination of grating and velocity, averaged over all experiments and window positions, versus the width of the observation window. Errors for *f*_FFT_ (blue line) refer to the initial guess obtained through naïve Fourier analysis, while the ones for *f*_LSq_ (green line) are related to the estimates gained by the minimization of Equation (9).

Figure 6 shows a graphical representation of the fitting procedure using the maximum allowed time window for the considered run. Such figure clearly shows the retrieval of the principal frequency coming out from the used combination of stimulation parameters. The frequency estimate errors, defined in Section 3.3 for comparing the FFT results with the fitting procedure described at the end of Section 3.2, are shown in Figure 7 and Table 3. Results from all windows and experiments were averaged for each point in the graphs.

As expected, errors in the initial guess *f*_FFT_ ranged from about $\frac{1}{2\text{w}}$ to almost zero, as the pattern of FFT-frequencies moved over the nominal frequency for each setup. However, the second estimation step using the least squares fit was very stable and converged for almost all windows larger than 0.4s. Using the entire available time series for each experiment, about 1.5s to 5s, leaded to the average errors shown in Table 3. To check consistency, results using piezoresistor 1 from MEMS sensors 1 and 2 in Equation (9) were compared to those using piezoresistor 1 from MEMS sensors 2 and 4. The errors obtained seem to agree very well, as shown in Table 3.

### Repeatability

**Figure 8.**
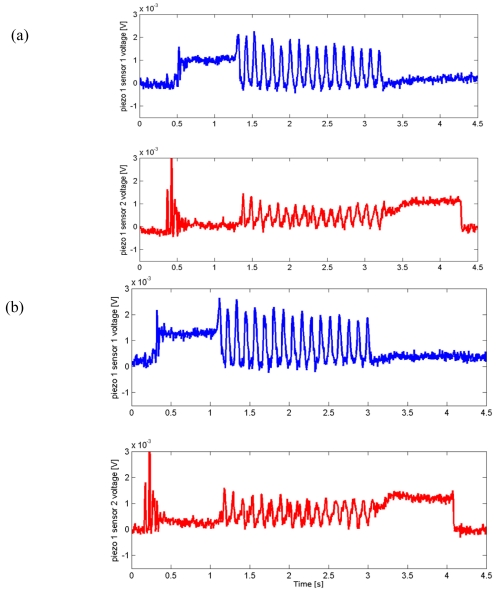
Time plot of the readings from piezoresistor 1 of MEMS sensors 1 and 2 of the array. The plots refer to data series 6 (a) and 7 (b), where a grating of 3.6 mm spatial periodicity was applied with translational speed of 30 mm/s, according to Tables 2 and 3. It is noticeable to observe the high repeatability, as well as the similarity with Figure 9(a), which only differs for the stimulus translational speed and thus results in an expansion of the time scale during phase C.

Figure 8 shows plots of the same channels within different runs having the same experimental conditions. Moreover, the cross-correlation coefficients defined in Section 3.3 confirmed a high degree of repeatability within one set of parameters *C*.

**Figure 9.**
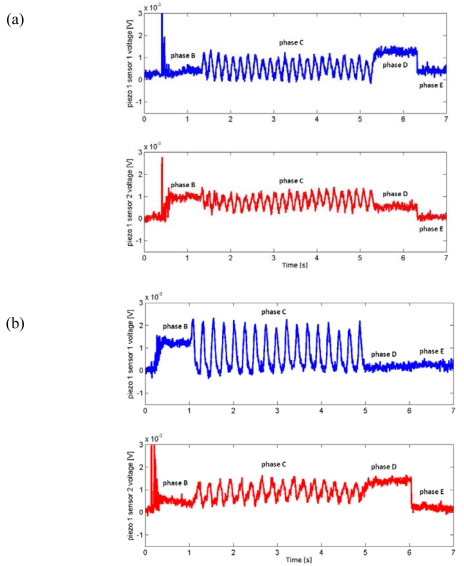
Time plot of the readings from piezoresistor 1 of MEMS sensors 1 and 2 of the array. The plots refer to data series 1 (a) and 31 (b), where gratings of 3.6 mm and 2.6 mm spatial periodicity were applied with translational speed of 15 mm/s, respectively, according to Tables 2 and 3. The frequency modulation due to the variation of the stimulus can be easily appreciated. The steps corresponding to the loading and unloading of the stimulus (phases A-B and D-E) may be more or less evident in a specific unit of the array depending on whether the ridge of the stimulus falls under a sensor unit or not, showing the static imaging potentiality of the tactile sensor array.

As an example, for piezoresistor 1 of MEMS sensor 1 the average Pearson correlation coefficients ranged from 0.87 to 0.97, while for piezoresistor 1 of MEMS sensor 2 their values went from 0.80 to 0.89 depending on the chosen parameters combination *C*. Moreover, all the coefficients for the channels close to the leading edge (e.g. MEMS sensor 1) of the stimulus during the sliding motion (phase C) were always higher than the ones for the channels at the falling edge (e.g. MEMS sensor 2) of the stimulus; this phenomenon is discussed in Section 5. The frequency modulation due to the variation of the stimulus can be appreciated in Figure 9, while a comparison between Figure 8 and Figure 9(a) points out the effect of stimulus velocity variation.

### Static Imaging

In parallel to the analysis of the frequency shift due to the variation of dynamic stimulation conditions, another major experimental result concerned the static imaging capabilities of the developed tactile sensor array. This further outcome was possible by choosing a proper experimental protocol, which included static phases in the initial and final parts of stimulation with repeatable conditions overall the runs. Figures 3, 8 and 9 show such results.

## 5. Discussion

The experimental results shown for dynamic artificial touch with medium-coarse periodic gratings demonstrated the perfect coherence between the principal frequency commonly revealed by the packaged MEMS sensor units and the expected one, as shown in Table 3 and Figure 7, as well as the consistency between the surface geometry and the static image of the stimulus-sensor interface.

Looking at the background of neurophysiological and psychophysical touch studies briefly reported in the Introduction, the technological and the signal processing outcomes of this work may be classified as a successful preliminary attempt to artificially achieve roughness encoding in case of medium-coarse patterning, i.e. a deterministic link (see Table 3) was obtained between the “spatial coarseness” of the presented stimuli and the features extracted from the sensor outputs.

These results pointed out the better processing quality guaranteed by using structured information from different units of a tactile sensor array instead of naïve Fourier analysis separately on each channel, overcoming frequency discretization limitations. These limitations are shown in Figures 4 and 5, which differ both in the time window length used for FFT and in the grating periodicity. The latter is the reason for the 1.4 Hz difference between the nominal frequencies, which could not be detected with FFT due to the low resolution of the FFT in the relevant frequency range (width of bars ∼0.5 Hz in Figure 4 *vs.* ∼2.5 Hz in Figure 5). As a consequence, using the naïve FFT approach to retrieve the frequency in a 0.35 s time window for both data series, would result in the two gratings being not distinguished, as shown in the respective plots of Figure 7. On the contrary, with the least squares fitting procedure the separation was well feasible, and the common frequency expected when indenting and sliding at constant speed periodic ridged surfaces across an array of sensors was accurately estimated. The technological approach together with the proposed frequency estimation method guaranteed an error from 1.7% down to 0.5% over the range of spatial frequencies considered, independently of the combination of MEMS sensor units used (see Table 3). Moreover, as shown in Figure 7, limiting the evaluation to fixed size time windows reduced the accuracy somewhat, but the method stayed stable down to 0.4 s window size, making it potentially suitable for most near real-time settings. The applied method revealed to be robust even if, in addition to the observable principal frequency shift associated to the combination of the used grating and stimulus sliding velocity, the signal power had overtones (the first three or four harmonics of the fundamental frequency) introduced by both the non-linear packaging and the sharp edges of the periodic ridged surfaces. On the contrary, the fitting based on Fourier analysis resulted in an oscillatory behavior of the error respect to the observation window length. Further stability and precision with the gradient descent fitting method could be gained by taking into account all four MEMS sensors and tuning the sampling rate according to the target application.

As depicted in Figure 8 and confirmed by the calculated average Pearson cross-correlation coefficients, the gathered data had high repeatability across different runs of the same experimental conditions. Furthermore, as seen in Figure 8 already, MEMS sensor 1 produced higher voltage amplitudes, leading to a better Signal-to-Noise (S/N) ratio, which in turn caused the higher correlation between runs with respect to MEMS sensor 2. This effect may be associated to the shape of the compliant packaging, which could induce higher stresses in piezoresistors of the sensor unit located at the leading edge. Reversing the scan direction (not shown) exchanged the roles of MEMS sensors 1 and 2 in this regard. Moreover, it is noticeable to observe the excellent similarity between the plots shown in Figure 8 and the plot of Figure 9 (a). These graphs only differ for the stimulus translational speed and thus result in a compression in the time scale during phase C.

**Figure 10.**
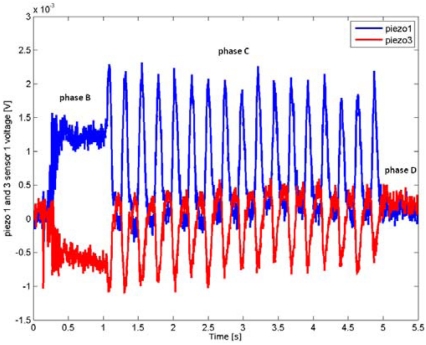
Time plot showing the opposite sign of the variation of the readings from piezoresistors 1 and 3 of MEMS 1 during sliding. The plot refers to data series 1, where a grating of 3.6 mm spatial periodicity was applied with translational speed of 15 mm/s, according to Tables 2 and 3.

The modulation of the principal frequency due to the variation of the stimulus can be appreciated in the time domain plots of Figure 9. Moreover, as detailed in Figure 10, couples of piezoresistors of a sensor unit which are located one in front to the other along the direction of motion [piezoresistors 1 and 3 in Figure 1(b)] responded with opposite sign to the stimulus. Therefore, even with packaged silicon sensors and dynamic stimulations, the symmetries of the static calibration matrix observed in [30] for the bare MEMS sensor were still present in this work.

Finally, as regards the consistency between the surface geometry and the static artificial touch representation, it is remarkable to observe the output signals variations relatively to the steps between phases A (starting of data acquisition) and B (sensor loading) and between phases D (steady state after stimulus sliding) and E (sensor unloading). Figures 3, 8 and 9 point out that the step heights varied between different runs depending on the used grating (but not on the velocity). This was due to the fact that a variation of the grating periodicity modified the portion of the ridge under each MEMS unit, being the initial and final position of the stimulus carrier always the same for all runs during phase C.

## 6. Conclusions and Future Work

The experimental analyses performed in this work demonstrated the suitability of the developed tactile sensor for revealing medium-coarse spatial features of the explored surface, both with dynamic and static stimulation. Future work will focus on performing similar experiments incorporating the developed technology in a bio-inspired mechatronic finger. Given that the feasibility of using a polyimide thin protective layer has been shown in this investigation, the actuated finger may use a polyimide glove (mimicking the human stratum corneum) for preventing the sensor to be worn or damaged by water or grit. Moreover, because of the possibility to integrate the readings from the array with proprioception information during active touch tasks, the combination of information regarding the estimated common frequency and the velocity of the finger could solve Equation (1) and provide quantitative measurements revealing texture properties of the explored stimuli. Investigations will also be performed in implementing processing strategies to separate the velocity and periodicity information contained in Equation (1) directly from the measurements of the array, thus avoiding the need to use the knowledge of the stimulus sliding velocity (in case of passive touch experiments) or proprioception information from an actuated finger (in case of active touch ones). Experiments will be performed with other stimuli, addressing not only a medium-coarse spatial periodic pattering, but also more general fine textures (e.g. sandpapers, gratings with oblique or aperiodic ridges or 2D patterning, …) and the frequency content due to the kind of material. In that case, the focus could move from principal frequency analysis to spectral analysis over the full frequency range, or to wavelet transform if the frequency content is supposed to change with respect to time and/or stimulus-sensor relative positioning. Moreover, the fact that the MEMS sensor is triaxial may be exploited in future work with stimuli having 2D patterning: in this paper, the raw sensor outputs were directly analyzed guaranteeing great accuracy in principal frequency retrieval without encoding the force vector at MEMS-packaging interface or at packaging-stimulus interface.

These planned experiments will require some modifications to the packaging design (e.g. lower thickness, material with different hardness or viscosity, introduction of fingerprints, etc.) in order to achieve even a higher sensitivity and selectivity for each MEMS unit [31,34] and a reduction of the low-pass spatial filtering effect introduced by the materials embedding the sensor [35] while still providing robustness for application in artificial hands dexterously interacting with the environment [29,32].

Finally, future investigations will experiment with artificial tactile sensors the unified paradigm proposed by Yoshioka and colleagues [13] for the perception of fine and coarse textured surfaces, in order to go towards a common theory for human and robot mediated coding and decoding of tactile stimuli.

## Figures and Tables

**Table 1.** Grating groove width (*gw*) and spatial period (Δ*p*) with respect to the sample type. Ridge width (*rw*) was fixed to 0.6 mm for all types.

**Grating number**	1	2	3	4
**gw (mm)**	2.0	2.5	3.0	3.5
**Δ*p* (mm)**	2.6	3.1	3.6	4.1

**Table 2.** Expected principal frequency from sensor outputs depending on the spatial periodicity (Δ*p*) and on the sliding velocity (*v*) of the applied grating.

**Expected frequency vs. Δ*p* and *v***	Δ*p* = 4.1 mm	Δ*p* = 3.6 mm	Δ*p* = 3.1 mm	Δ*p* = 2.6 mm
*v* = 15 mm/s	3.66 Hz	4.17 Hz	4.84 Hz	5.77 Hz
*v* = 30 mm/s	7.32 Hz	8.33 Hz	9.68 Hz	11.54 Hz
*v* = 48 mm/s	11.71 Hz (not tested)	13.33 Hz (not tested)	15.48 Hz	18.46 Hz

**Table 3.** Average RMS errors obtained by FFT (ε_FFT_) and least square fit estimation (ε_LSq_) using the full data range. The top row indicates the expected frequency, *f_nominal_*, depending on the measurement run. The combination *C* of spatial period Δ*p* and velocity *v* associated to each measurement run is also indicated.

	**Measurement run**									
	**1-4**	**5-8**	**9-12**	**13-16**	**17-20**	**21-24**	**25-28**	**29-32**	**33-36**	**37-40**
***f****_nominal_***[Hz]**	4.17	8.33	3.66	7.32	4.84	9.68	15.48	5.77	11.54	18.46
***C***	C_1_	C_2_	C_3_	C_4_	C_5_	C_6_	C_7_	C_8_	C_9_	C_10_
**Δ*p* [mm]**	3.6	3.6	4.1	4.1	3.1	3.1	3.1	2.6	2.6	2.6
***v* [mm/s]**	15	30	15	30	15	30	48	15	30	48

	**piezo_1,1_ vs. piezo_2,1_**									

**ε_FFT_**	0.072	0.135	0.113	0.182	0.078	0.168	0.286	0.033	0.053	0.063
**[%]**	1.72	1.62	3.1	2.49	1.62	1.73	1.85	0.57	0.46	0.34
**ε_LSq_**	0.038	0.105	0.027	0.058	0.052	0.123	0.257	0.053	0.124	0.272
**[%]**	0.91	1.26	0.73	0.79	1.08	1.27	1.66	0.93	1.07	1.47

	**piezo_4,1_ vs. piezo_2,1_**									

**ε_FFT_**	0.072	0.135	0.1	0.152	0.075	0.165	0.257	0.036	0.059	0.189
**[%]**	1.72	1.61	2.74	2.08	1.56	1.7	1.66	0.62	0.52	1.03
**ε_LSq_**	0.04	0.127	0.029	0.063	0.06	0.126	0.262	0.055	0.123	0.266
**[%]**	0.97	1.52	0.,78	0.87	1.24	1.31	1.69	0.95	1.07	1.44
